# 4341 Neuroclinical fingerprints of risk for psychosis: Profiles of neurophysiology, symptom severity, and cognitive function

**DOI:** 10.1017/cts.2020.416

**Published:** 2020-07-29

**Authors:** Keisha Novak, Sam Buck, Roman Kotov, Dan Foti

**Affiliations:** 1 Purdue University; 2 Stony Brook University

## Abstract

OBJECTIVES/GOALS: The study aims to utilize event-related potentials (ERPs) coupled with observable reports of symptoms to comprehensively understand neurological and symptomatic profile of individuals at risk for developing psychosis. The study is a short-term longitudinal design which allows for examination of course as well as structure of illness. METHODS/STUDY POPULATION: This study uses a combination of well-validated ERPs (P300, N400, ERN) and symptom data to predict variation in symptoms over time. We parse heterogeneity within a high-risk group to create innovative profiles and predict variation in course of symptoms. Data collection is ongoing (n = 35; target N = 100). Methods include a battery of ERP tasks tracking neural processes associated with attention, language processing, and executive function (P300, N400, ERN), along with assessment of symptom type and severity. Analyses include how ERPs correlate with severity of risk and symptom dimensions (positive, negative, disorganized). We examine whether individual versus global ERP aberrations (P300, N400, ERN) predict individual versus global symptom domain severity (positive, negative, disorganized), or vice versa. RESULTS/ANTICIPATED RESULTS: Symptom domain scores were elevated compared to general population on positive (*M* = 1.65, *SD* = .36), negative (*M* = 1.9 *SD* = .42), and depressive (*M* = 1.94, *SD* = .40) domains. Small to medium effect sizes emerged for P300 profile (r’s = −.001 to −.41) and ERN profile (r’s = −.03 to −.37), though small effect sizes for N400 profile (r’s = −.06 to .29). Analyses were run to determine the degree to which profiles of risk were similar: P300/ERN (r = −.09), ERN/N400 (r = −.39), and N400/P3 (r = −.20). Additional analyses suggest potential mediating effects of cognition on neural activity and symptoms. DISCUSSION/SIGNIFICANCE OF IMPACT: We use a combination of well-validated ERPs (i.e. P300, N400, ERN) with behavioral and symptom data to predict variation in symptoms over time. A “fingerprint” physiologic aberration may be exhibited within high-risk individuals and can be used as biomarkers to identify those at risk even before onset of observable symptoms.

